# Self-Supervised Plant Phenotyping by Combining Domain Adaptation with 3D Plant Model Simulations: Application to Wheat Leaf Counting at Seedling Stage

**DOI:** 10.34133/plantphenomics.0041

**Published:** 2023-04-11

**Authors:** Yinglun Li, Xiaohai Zhan, Shouyang Liu, Hao Lu, Ruibo Jiang, Wei Guo, Scott Chapman, Yufeng Ge, Benoit Solan, Yanfeng Ding, Frédéric Baret

**Affiliations:** ^1^ Plant Phenomics Research Centre, Academy for Advanced Interdisciplinary Studies, Jiangsu Collaborative Innovation Center for Modern Crop Production, Nanjing Agricultural University, Nanjing, China.; ^2^ Key Laboratory of Image Processing and Intelligent Control, School of Artificial Intelligence and Automation, Huazhong University of Science and Technology, Wuhan, China.; ^3^ Graduate School of Agricultural and Life Sciences, The University of Tokyo, 1-1-1 Midori-cho, Nishitokyo City, Tokyo, Japan.; ^4^ School of Agriculture and Food Sciences, The University of Queensland, St. Lucia, Queensland 4072, Australia.; ^5^ Department of Biological Systems Engineering, University of Nebraska-Lincoln, Lincoln, Nebraska 68583, United States.; ^6^ INRAE, Avignon Université, UMR EMMAH, UMT CAPTE, 228, route de l’aérodrome - CS 40509, 84914 Avignon Cedex 9, France.; ^7^ ARVALIS Institut du végétal, 3 rue Joseph et Marie Hackin, 75116 Paris, France.

## Abstract

The number of leaves at a given time is important to characterize plant growth and development. In this work, we developed a high-throughput method to count the number of leaves by detecting leaf tips in RGB images. The digital plant phenotyping platform was used to simulate a large and diverse dataset of RGB images and corresponding leaf tip labels of wheat plants at seedling stages (150,000 images with over 2 million labels). The realism of the images was then improved using domain adaptation methods before training deep learning models. The results demonstrate the efficiency of the proposed method evaluated on a diverse test dataset, collecting measurements from 5 countries obtained under different environments, growth stages, and lighting conditions with different cameras (450 images with over 2,162 labels). Among the 6 combinations of deep learning models and domain adaptation techniques, the Faster-RCNN model with cycle-consistent generative adversarial network adaptation technique provided the best performance (*R*^2^ = 0.94, root mean square error = 8.7). Complementary studies show that it is essential to simulate images with sufficient realism (background, leaf texture, and lighting conditions) before applying domain adaptation techniques. Furthermore, the spatial resolution should be better than 0.6 mm per pixel to identify leaf tips. The method is claimed to be self-supervised since no manual labeling is required for model training. The self-supervised phenotyping approach developed here offers great potential for addressing a wide range of plant phenotyping problems. The trained networks are available at https://github.com/YinglunLi/Wheat-leaf-tip-detection.

## Introduction

Early seedling vigor is an important trait used to evaluate crop implantation and affect its future development [1–3]. In cereal crops, the dynamics of the leaf number per plant informs about the phenological stage [4]. It is used to compare the growth rate between cultivars, cultural practices, and environments and then to guide farmers on the optimal cultivation methods, such as irrigation and fertilization [5]. Conventional methods for measuring the leaf number in the field consist of directly counting the leaves on a sample of plants. This process is very slow and labor-intensive and is generally associated with large uncertainties because of the small samples used [6,7]. It is difficult to get accurate estimates of some traits derived from leaf counting, such as growth rate or the phyllochron [4,8–10]. The image-based high-throughput phenotyping provides a potential alternative to the conventional leaf counting methods that are low-throughput, intrusive, and sometimes even destructive [11–13]. Deep learning techniques are now routinely exploited to extract specific traits from high-resolution images [14–16]. The objective of this study is to develop a high-throughput method for leaf tip counting from RGB images taken in the field.

Deep learning has been developed mainly using object detection [17,18] or segmentation [19] algorithms. They have been developed to estimate the number of plants or organs (leaves, spikes, etc.)[20], the canopy cover or the green cover [21] , and the emergence rate [22]. Detection algorithms have already been used to count the number of leaves [23]. Leaf tips are specific features of the leaves well adapted for leaf detection and localization. However, leaf tips are typical “tiny objects” in images, which is a difficult problem in the field of object detection [24]. As a result, existing research on leaf tip detection has been carried out mainly under controlled conditions in greenhouses [23,25]. The sparse canopy structure, complex soil background, and small size of leaves require a very large annotation workload to achieve accurate enough leaf counts in the field [26]. Further, monitoring the dynamics of the leaf number requires an even larger amount of highly accurate annotated images to train the deep learning model [27–29]. It is therefore highly desired to get effective methods for generating annotated training samples.

Synthetic images have already been used to solve data acquisition challenges while improving the accuracy and robustness of the algorithm [30–32]. Crop model simulations provide a potential way to tackle the image annotation problem [33]. Crop model could directly generate the annotated dataset required to train the deep learning model [31,34–36]. Unfortunately, crop models are often lacking realism, and deep learning models trained only with synthetic data fail to perform well when applied to actual images [37,38].

In deep learning, a domain refers to the feature space or the distribution of the data. It is the fact that the feature difference between the simulated dataset and the real dataset, i.e., domain gap, that causes the model trained on synthetic images cannot generalize well to the actual images. Domain randomization [37,39] and domain adaptation [40,41] techniques are widely used to further reduce the reality gap [42,43]. The digital plant phenotyping platform (D3P) [44] generates wheat crop images using a realistic 3-dimensional (3D) canopy structure model that simulates the 3D dynamics of wheat canopy structure based on the knowledge of several botanical processes [45]. D3P uses a physically based ray tracer to render images. It is also possible to use texture extracted from leaf and ground elements in actual images to improve the realism of the rendering and reduce the domain gap problem. Highly realistic training datasets can therefore be automatically generated by combining D3P model simulations and domain randomization or adaptation techniques, avoiding the laborious manual annotation process that may be also prone to errors. Generative adversarial networks (GAN) are now widely used for domain adaptation, such as cycle-consistent GAN (CycleGAN) [46]. However, CycleGAN strategies require already a high degree of consistency between the simulated and the actual images [47], which place demands on the methods to generate simulation data.

This study aims to develop a leaf tip counting method from RGB images of the crop under field conditions based on deep learning approaches where the training dataset is generated by combining RGB image simulations and CycleGAN domain adaptation techniques. A large dataset of wheat crop RGB images taken in the field under contrasted conditions was collected. A subsample of these images was manually labeled to evaluate the performance of the proposed method. Further, the factors controlling the realism of the simulated images, the impact of the spatial resolution, and the efficiency of CycleGAN domain adaptation technique were analyzed.

## Materials and Methods

### Building the actual RGB images dataset of wheat crops at early stages

We collected 2,763 RGB images of wheat field at juvenile stages over 11 locations distributed within 5 countries (China, France, Japan, United States, and Australia) (Table 1). We use the Haun scale to quantitatively describe wheat plant development. It corresponds to the number of fully expanded leaves. This dataset covers wheat development mainly between Haun stages 1 and 4 before tillering. The images were captured using different types of cameras, from high-quality single-lens reflex camera to portable action camera or smartphone. In addition, they were taken from 0^o^ to 45° view zenith angle, with a spatial resolution varying between 0.1 and 0.5 mm per pixel. Images were taken either from a camera on a fixed pole in the field, using hand-held systems, or using a ground vehicle. The dataset covers a wide range of variability with respect to the canopy structure, soil background, and light conditions. A 1,024 × 1,024 size window was cropped in the center of the images to model training. In the following, we will only consider the 1,024 × 1,024 images. All these images were manually labeled for model training or testing. A total of 1,600 of these images will be involved in domain-adaptive training, and the remaining images will be used for testing or independent testing.

**Table 1.** Detailed description of the RGB wheat images dataset used for model training and testing.

Country	Sites	Latitude	Longitude	Camera	Field of view	Ground sampling distance (mm per pixel)	Image size (pixels)	Number of images	Shooting mode	Note	Vector
China	Yangling	34.3°N	108.1°E	HUNTERCAM_H881	45°	0.3–0.4	4,068 × 3,456	73	Fixed	Testing	Fixed pole
	Jinan	36.4°N	117.0°E	HUNTERCAM_H881	45°	0.3–0.4	4,068 × 3,456	60	Fixed	Testing	Fixed pole
	Xinxiang	35.2°N	113.6°E	HUNTERCAM_H881	45°	0.3–0.4	4,068 × 3,456	60	Fixed	Testing	Fixed pole
	Xuzhou	34.2°N	117.1°E	SONY RX0 II	45°	0.3–0.4	5,472 × 3,648	689	Automatic	Training	Handheld system
	Jurong	31.9°N	119.2°E	SONY RX0 II	45°	0.3–0.4	5,472 × 3,648	309	Automatic	Training	Handheld system
	Baima	31.6°N	119.2°E	SONY RX0 II	0°,45°	0.3–0.4	5,472 × 3,648	1,385	Automatic	Training	Handheld system
France	Toulouse	43.5°N	1.5°E	Sigma SD14	45°	0.2–0.3	4,068 × 3,072	40	Automatic	Testing	Handheld system
	Paris	48.8°N	1.9°E	NIKOND 5200	45°	0.1–0.2	6,000 × 4,000	20	Automatic	Testing	Handheld system
	Avignon	43.9°N	4.8°E	Sigma SD14	45°	0.1–0.2	4,068 × 3,072	27	Automatic	Testing	Handheld system
Australia	Queensland	27.4°N	153.0°E	GECKO-CAM	45°	0.4–0.5	4,032 × 3,024	40	Fixed	Testing	Fixed pole
Japan	Tokyo	35.4°N	139.4°E	Canon EOS X5	0°,45°	0.1–0.2	5,184 × 3,456	30	Fixed	Testing	Fixed pole
United States	Lincoln, Nebraska	40.8°N	96.7°W	Smart Phone	45°	0.1–0.2	3,024 × 4,032	30	Automatic	Testing	Handheld system

**Fig. 1.**
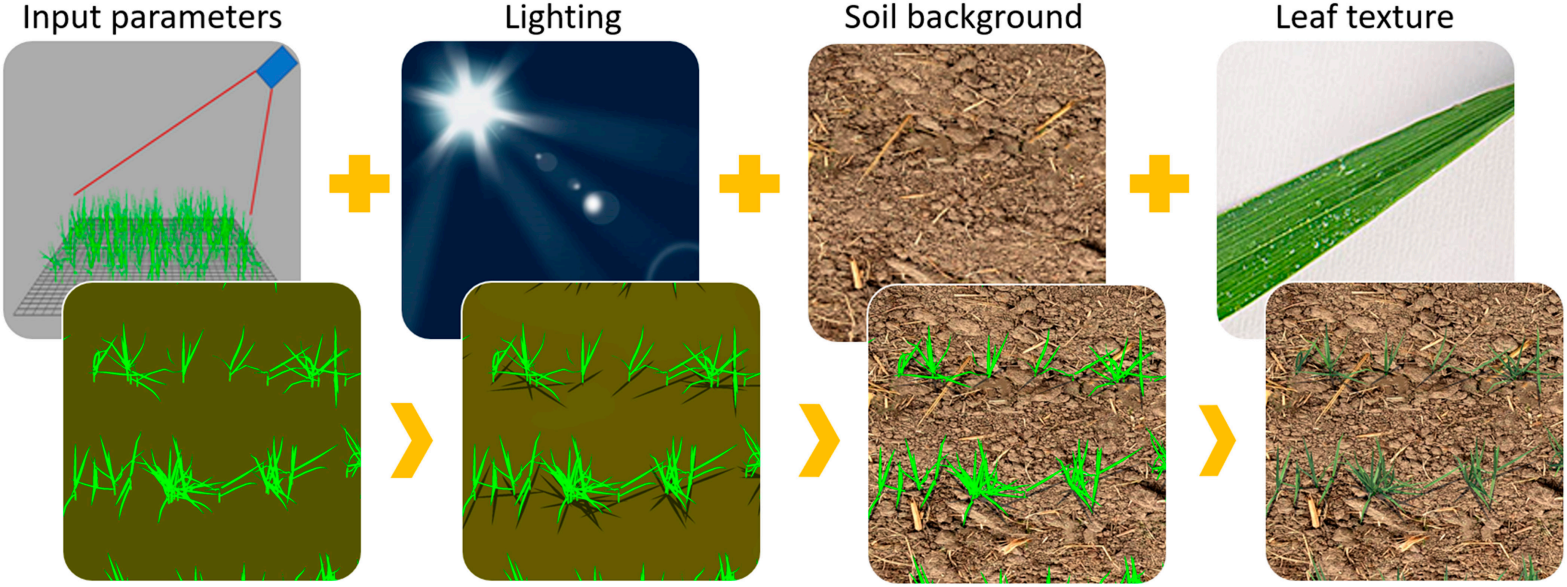
Simulating field wheat images with D3P. From left to right, through accounting for more and more factors in the simulation pipeline using D3P (in the top column), the simulated image would turn to be more and more realistic (in the bottom column).

### Generating simulated wheat images

We generated in total of 16,000 simulated wheat images with 2,461,503 leaf tips annotated automatically using the D3P developed in our previous work [44]. D3P consists of 2 parts: a functional–structural plant model (FSPM) to generate 3D realistic canopy structure and a physically based raytracer to simulate images over the virtual 3D canopies. We used the state of the art wheat FSPM, ADEL-wheat model [45]. It is implemented in the FSPM modeling platform, OpenAlea (http://openalea.gforge.inria.fr) [48]. Through manipulating the input parameters, D3P could simulate the dynamics of various canopy structures corresponding to different cultivars and field managements. It provides the 3D meshes of the virtual canopies. The open-source raytracing software POV-Ray 3.7 (www.povray.org) was then used to simulate the RGB images. The settings of the virtual camera were configured similarly to the ones used in our actual dataset (Table 1). POV-Ray can simulate images under different light conditions and with different optical properties of the leaf, stem, and soil backgrounds, including rendering the texture. Note that the soil background and leaf texture were added through projecting actual background and leaf images onto the corresponding object surface in the 3D scene (Fig. 1). Finally, D3P allows to play with 6 types of factors to simulate the synthetic images: (a) 3D canopy structures, including the parameters describing leaf dimensions, leaf inclination, sowing density, or tiller number; (b) development stages controlled by the cumulative thermal time after emergence; (c) leaf texture, manipulated using the scanned actual leaf images; (d) soil background, manipulated using actual bare soil images taken in the field; (e) light conditions, including parameters to describe the illumination geometry and the composition of direct and diffuse light; (f) configurations of the virtual camera including the image resolution, field of view, and viewing direction.

As suggested in our previous work [49], the camera configuration was fixed with 45^o^ viewing angle at 1.8 m in height with a 5,472-pixel × 3,648-pixel images. These original images were divided into 1,024-pixel × 1,024-pixel subimages using sliding windows with some overlap between consecutive subimages. We therefore considered only the 5 first factors to build the synthetic images dataset. We sampled more than 27 times from the parameter space using Latin hypercube sampling and 8 stages (including 227 parameters) [44]. We generated 15,000 images (1,024 × 1,024 subimages) using 40 h with our local computer (2× Titan V, 2× Intel Xeon Platinum 8180). Bounding boxes of 12-pixel × 12-pixel labels were automatically placed on the leaf tips for each image.

### Generating Sim2Real wheat images

**Fig. 2.**
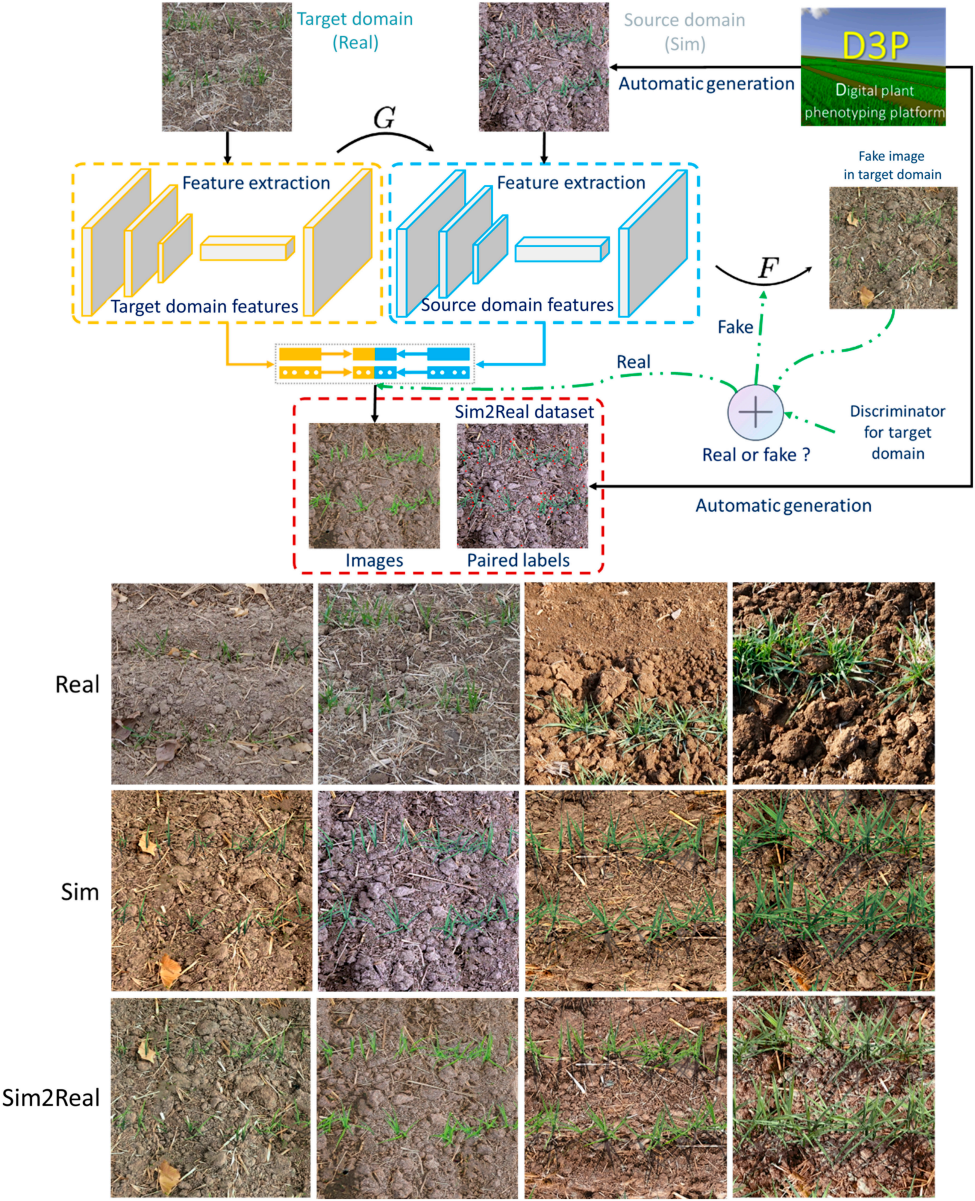
Translating the simulated wheat images (Sim dataset) into Sim2Real dataset using CycleGAN.

The CycleGAN [50] domain adaptation technique was selected to translate the source domain of the simulated dataset (X) into the target domain of the real images (Y). The translated dataset, including 16,000 images, would be called Sim2Real dataset hereafter. Basically, CycleGAN trains simultaneously the translator G: Real → Sim and another translator F: Sim → Real with a cycle consistency loss that encourages images generated in the source domain and is indistinguishable from images in target domain (Fig. 2). The simulation results of the source domain image are compared with the target domain image in each round. The training is not terminated until the Euclidean distance is less than 10 (see the Evaluation of the gap after domain adaptation section for details of the Euclidean distance). This is a long process, because, importantly, CycleGAN respects the boundary of leaves, consequently preserves the position of the leaf tips, and ensures that the labels generated automatically in the simulation process are valid for the Sim2Real images. The CycleGAN was implemented with default parameters (using the adversarial loss and consistency loss with 1.2 weight of size) for training, and 5,000 simulated and 5,000 actual 1,024 × 1,024 images were used as source and target domains, respectively.

### The convolutional neural networks models considered

We considered 6 states of the art object detection models for leaf counting (Table 2). The first 4 models were trained over 15,000 images from the Sim2Real dataset using the CycleGAN domain adaptation technique described earlier. Conversely, the last 2 models were trained over the Sim (15,000 images) and Real (1,500 images) datasets based on enhanced adaptation techniques. More detailed model description is given in the Supplementary Materials. To evaluate the possible added value of the domain adaptation techniques, we also trained the first 4 object detection models over the simulated dataset (Sim, 15,000 images) or actual dataset (Real, 1,500 images). The same set of hyperparameters was used for all the training modalities, and all the images in the dataset are the same size with 1,024 × 1,024.

**Table 2.** Object detection models and domain adaptation techniques used.

**Detection model**	**Domain adaptation**	**Model name**	**Training dataset**		**Reference**
			**Sim + Real**	**Sim2Real**	
YoloV5	CycleGAN	CG-YV5		**√**	[51]
FRCNN		CG-FRCNN		**√**	[52]
TasselNetV2+		CG-TNV2+		**√**	[20]
P2PNet		CG-P2P		**√**	[53]
FRCNN	H-divergence	DA-FRCNN	**√**		[54]
DETR	SFA	SFA	**√**		[26]

### Accuracy assessment

#### Evaluation of the gap after domain adaptation

To measure the differences between Sim and Real datasets, we visualization the domain gap between the datasets using the t-distributed stochastic neighbor embedding (t-SNE) [51]. The t-SNE is one of the most popular algorithms for dimensionality reduction of high-dimensional data. The similarity between data is expressed by transforming the high-dimensional Euclidean distance between data in a dataset into a conditional probability. In addition, we calculated the Euclidean distance between the datasets, which is calculated as follows:

$$\mathrm{Euc}.=\sqrt{\sum_{i=1}^{n}{\left(x_{i}-y_{i}\right)}^{2}}$$ (1)

#### Evaluation of deep learning models

The performances of the leaf counting models were evaluated in 2 phases: In the development phase, the accuracy of all the 6 models was evaluated over 400 images taken from the testing sites in China. This allowed selecting the best performing model that was then further evaluated over the other worldwide distributed testing sites presenting a large diversity of situations (Table 1).

We used the mean absolute error (MAE), root mean square error (RMSE), and *R*^2^ as assessment metrics. It is calculated using precision (*p*) and recall (*r*):

$$p=\frac{\mathrm{TP}\left(t\right)}{\mathrm{TP}\left(t\right)+\mathrm{FP}\left(t\right)}$$ (2)

$$r=\frac{\mathrm{TP}\left(t\right)}{\mathrm{TP}\left(t\right)+\mathrm{FN}\left(t\right)}$$ (3)

where *TP(t)* indicates the number of instances detected with an intersection over union (IoU) of >0.5; *FP(t)* is the number of instances detected with an IoU of ≤0.5 while corresponding to an actual instance; and *FN(t)* is the number of instances not detected. The MAE, RMSE, and *R*^2^ for leaf counting accuracy assessment were defined as follows:

$$\mathrm{MAE}=\frac{1}{n}\sum_{i=1}^{n}\left|y_{i}-{\hat{y}}_{l}\right|$$ (4)

$$\text{RMSE}=\sqrt{\frac{1}{n}\sum_{i=1}^{n}\left(y_{i}-{\hat{y}}_{l}\right)}$$ (5)

$$R^{2}=1-\frac{\sum_{i=1}^{n}{\left(y_{i}-y_{i}^{\prime }\right)}^{2}}{\sum_{i=1}^{n}{\left(y_{i}-\hat{y_{i}}\right)}^{2}}$$ (6)

where *n* denotes the number of objects to be compared, *y_i_* indicates the value of the manual measurement result, $\hat{y_{i}}\;$ denotes the values of the model, and $y_{i}^{\prime }$ indicates the mean of manual measurement results.

### Factors impacting the leaf counting models

We investigated the impact of some of the factors used to generate the simulated images on the leaf tip counting performances. This was done using the selected best performing model with evaluation over 400 actual images taken from the testing sites in China (Table 1).

#### Realism of the simulated dataset

Soil background, leaf texture, and illumination conditions are the main factors determining the realism of the Sim dataset. Subsequently, they may impact the efficiency of the domain adaption strategy and the performance of the model trained using Sim2Real dataset. To quantify this impact, we conducted in silico experiments with D3P by considering several combinations of these 3 factors: (a) leaf texture only, (b) illumination conditions only, (c) soil background only, (d) leaf texture and illumination conditions, (e) leaf texture and soil background, and (f) illumination conditions and soil background. When the factors are not considered, they are set to default values, i.e., set as the same standard soil background, the standard leaf texture, and the constant fully diffused illumination conditions. For each in silico experiments, 5,000 images were simulated to generate the Sim2Real dataset using CycleGAN along with the 1,500 real images.

#### Spatial resolution

The impact of the spatial resolution was evaluated using the best performing model and the most realistic simulated images (variation in soil background and illumination conditions while using realistic leaf texture). We simulated a set of wheat images with 0.1- to 2.0-mm spatial resolution by 0.1-mm steps. This was achieved by adjusting the height of the virtual camera (POV-Ray 3.7). The complete processing including CycleGAN and detection model training was applied for each spatial resolution image.

## Results

### Domain adaptation techniques drastically improve the capacity to detect leaf tips

When trained over the simulated dataset without any domain adaptation, all the 4 detection models considered are performing very well when applied to actual other simulated images (see Table S1). This demonstrates the capacity of these convolutional neural network (CNN) to identify well the leaf tips. However, when they are applied to actual images, they are performing very poorly (Table 3). A slight improvement is observed when the models are trained over the actual images (Table 3). Although the training dataset used for the actual images was limited (400 images as compared to 15,000 for the simulated dataset), the realism of the images appears to be a key feature when training for a relatively complex problem such as the leaf tip detection. The 4 detection models trained over the Real dataset perform similarly, with a small advantage for Point-to-Point Network (P2P) and Faster-Regions with Convolutional Neural Network Features (FRCNN). However, the best performances are observed when domain adaptation techniques are applied (Table 3), particularly for the latest stages when the leaf tip detection is more difficult.

**Table 3.** Performances of all the models for leaf tip detection and the counting. The colors indicate the goodness of the performances (dark green, the best; dark red, the poorest) for each metrics. All the models were evaluated over the Chinese dataset (400 images).

	**No domain adaptation**						**Domain adaptation**			
**Training dataset**	**Sim**			**Real**				**Sim2Real**		
**Detection model**	**MAE**	**RMSE**	***R*** ^**2**^	**MAE**	**RMSE**	***R*** ^**2**^	**Model**	**MAE**	**RMSE**	***R*** ^**2**^
YV5	—	—	—	18	25.4	0.51	CG-YV5	16.4	23.8	0.68
FRCNN	25.7	34.8	−3.30	14.1	19.0	0.61	CG-FRCNN	6.0	8.7	0.94
TNV2+	35.8	43.8	−0.90	16.0	22.3	0.58	CG-TNV2+	14.9	20.3	0.74
P2P	47.4	59.8	−1.10	15.5	23.5	0.68	CG-P2P	7.2	9.8	0.90
							DA-FRCNN	15.9	21.1	0.66
							SFA	10.3	13.4	0.81

**Fig. 3.**
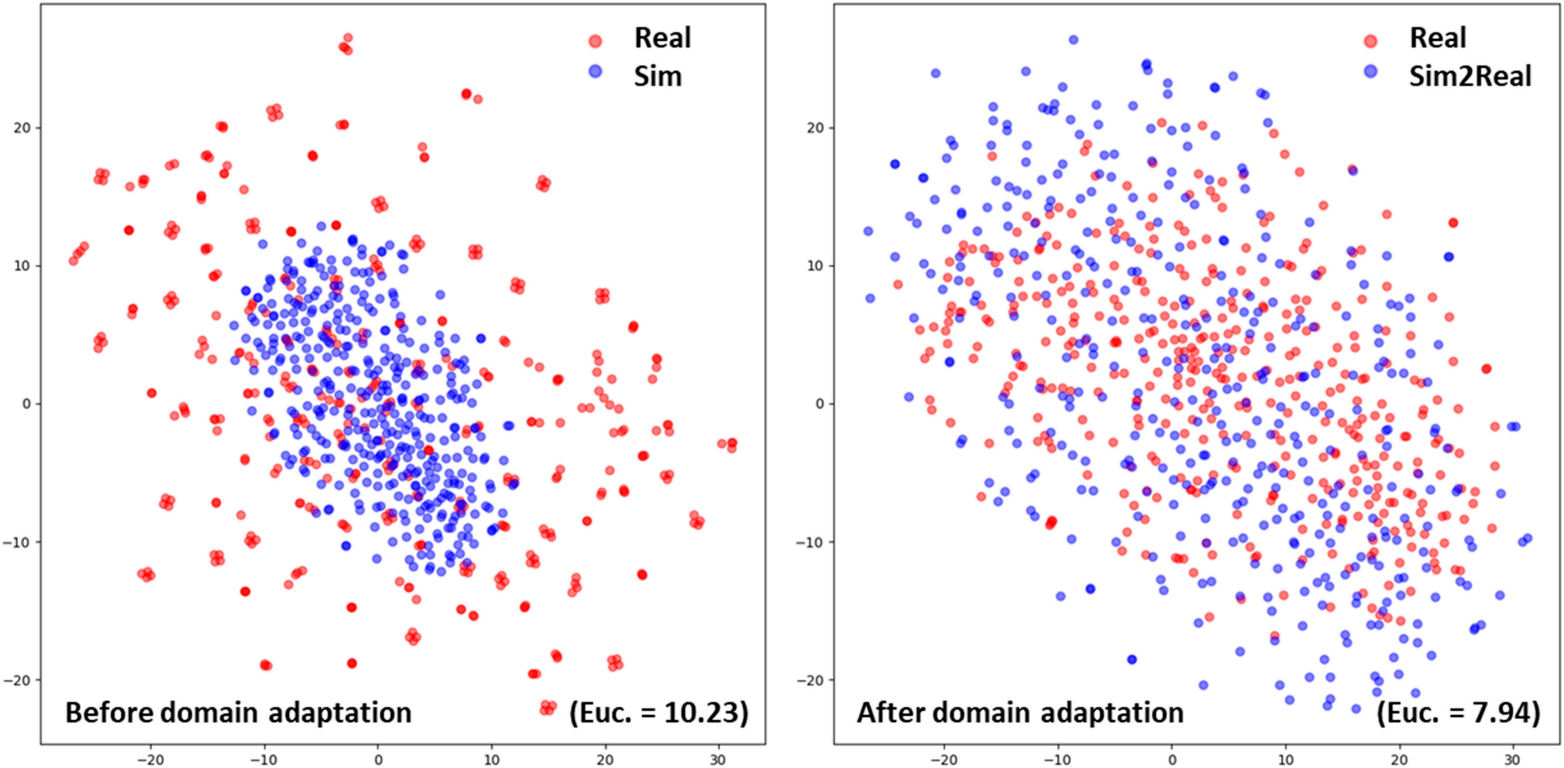
Distribution of the features learned using a model trained over a given dataset using the t-SNE technique. On the left, the features extracted from the Sim and Real datasets are presented. On the right, the features extracted from the Sim2Real and Real datasets are presented.

We used t-SNE to visualize the features learned after training the FRCNN model with the 3 datasets. The features are mainly derived from the output of the Region Proposal Network (RPN). RPN is the main feature extraction network for FRCNN. Therefore, the features from RPN layer are representative. The features extracted over the Sim dataset only capture a small part of those extracted over the Real dataset (Fig. 3). Conversely, when CycleGAN is applied to the simulated images to generate the Sim2Real dataset, the features extracted are much closer. The disparity between the features extracted by the several models was quantified using the Euclidian distance, *d*. The distance is reduced for the domain adapted training dataset [*d*(Real, Sim2Real) = 7.9] as compared to the raw simulated dataset [*d*(Real, Sim) = 10.2], demonstrating the efficiency of the CycleGAN domain adaptation technique.

### CG-FRCNN outperforms the other models

**Fig. 4.**
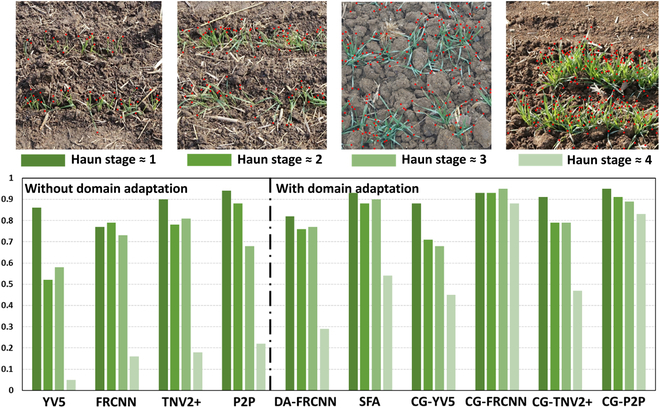
Performances of the models as a function of the development stage. When no domain adaptation is applied, the results displayed correspond to models trained with the real dataset.

The CycleGAN (CG)-FRCNN model trained using the Sim2Real dataset performs the best (RMSE = 8.7, *R*^2^ = 0.94), providing very consistent accuracy from Haun stages 1 to 4 (Table 3 and Fig. 4). Conversely, domain adaptation (DA)-FRCNN performs the worst among the 6 models with domain adaptation. These 2 models use the same FRCNN object detector but differ in the domain adaptation technique. This demonstrates that both the detection model and domain adaptation strategies are important for the success of the approach developed here. While all the models perform relatively accurately for the earliest stages (Fig. 4), most of the differences are observed for the latest stages where the identification problem is more difficult because of partly overlapping leaves (Fig. 4, top). In this case, the domain adaptation strategy remarkably improves the overall accuracy of leaf counting, due to its success in enriching the training dataset while keeping a high level of realism.

### Impact of the realism on leaf tip detection and counting performances

The impact of 3 main factors used to render realistic images including light condition, leaf texture, and soil background was evaluated on the performances of leaf tip detection and counting. This was achieved using the CG-FRCNN model that was previously demonstrated to be the most accurate one (Table 3 and Fig. 4). Performances were evaluated over the 400 test images extracted from the China dataset (Table 1).

**Fig. 5.**
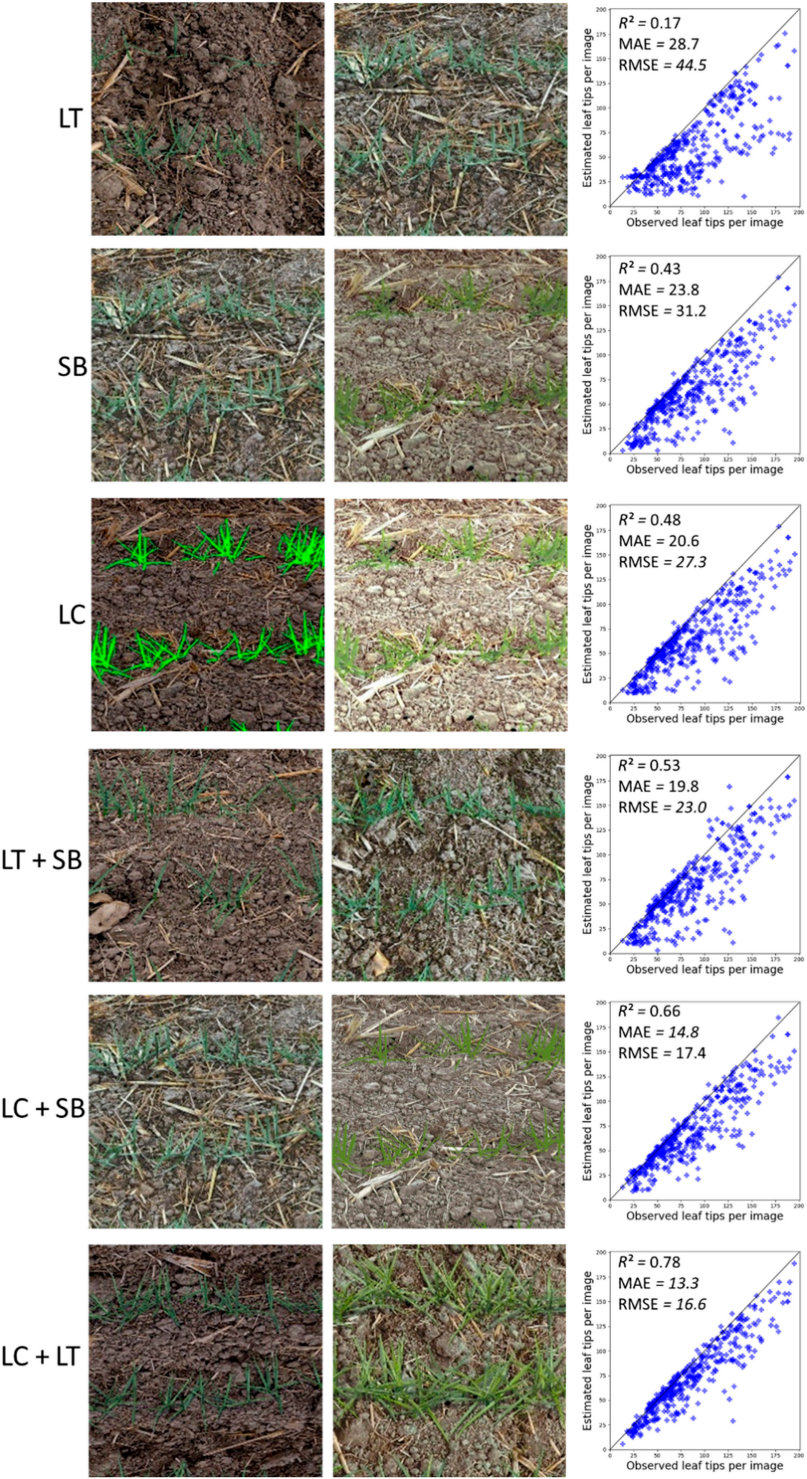
Visualisation results of different domain adaption strategies and the performance of the mode trained using Sim2Real dataset.

Results show that the light condition is the most important single factor to account for, while adding leaf texture is the less important one (Table 4). Most of the problems occur for the latest Haun stages with a clear underestimation of the leaf tip numbers (Fig. 5), i.e., leaf tips not detected by the model. The realism of the simulated images could be also well quantified using the Euclidian distance computed on the t-SNE feature plane (Table 4). CycleGAN adaptation domain technique appears more efficient when the simulated images have already a high degree of realism.

**Table 4.** Performance of the leaf counting model trained depending on the degree of realism of the simulations. Three single factors and their combinations were considered in the generating of the Sim2Real dataset, including leaf texture (LT), soil background (SB), and light condition (LC). *d* means the Euclidean distance. For each metrics, the colors indicate the goodness (dark green, the best; dark red, the poorest). In this experiment, we considered all stages. Thus, each factor contains 4 stages of wheat images.

**Factors**	**RMSE**	***R*** ^**2**^	***d***(**Real**, **Sim**)	***d***(**Real**, **Sim2Real**)
LT	44.5	0.17	22.96	21.34
SB	31.2	0.43	20.12	18.78
LC	27.3	0.48	19.86	16.66
LT + SB	23	0.53	17.83	15.86
LC + SB	17.4	0.66	14.37	12.52
LC + LT	16.6	0.78	10.41	9.23
LC + LT + SB	8.7	0.94	10.23	7.94

When considering the combination of 2 factors, leaf texture and soil background provide the lowest realism, confirming that light conditions is the most important single factor. Light conditions interact with the shadows of the leaves that add a high degree of complexity in the images. Best realism is obtained by combining light conditions and leaf texture (Table 4). However, the combination of the 3 factors together still improves the realism (Euclidian distance) and the overall performances (RMSE and *R*^2^), with no systematic underestimation of the leaf tip number.

### Impact of the spatial resolution on the performances

**Fig. 6.**
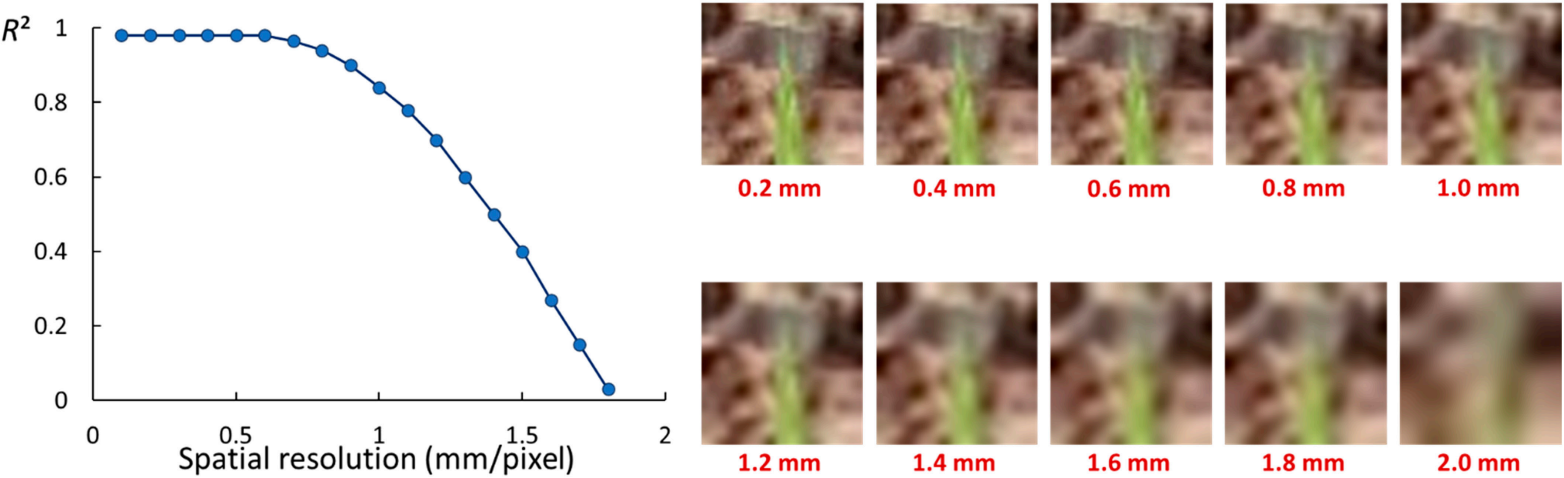
The impact of different spatial resolutions on the estimation accuracy (left). On the right, a simulated image with different spatial resolutions.

We investigated here the role of the spatial resolution of the simulated images on leaf tip detection and counting performances. The CG-FRCNN model was used, with the best realism for image rendering. Results show that the estimation accuracy stays at its maximum up to a spatial resolution of 0.6 mm per pixel (Fig. 6, left). This corresponds roughly to the lowest resolution of the actual images of the test dataset (0.4 mm per pixel; see Table 1) and to the resolution required to clearly identify the leaf tips (Fig. 6, left). When the size of the pixels increases, the performances degrade quickly, with even no capacity to detect leaf tips for resolution larger than 1.7 mm per pixel, when it is even impossible to identify them visually (Fig. 6, right).

### The model CG-FRCNN is robust across a large range of conditions

**Fig. 7.**
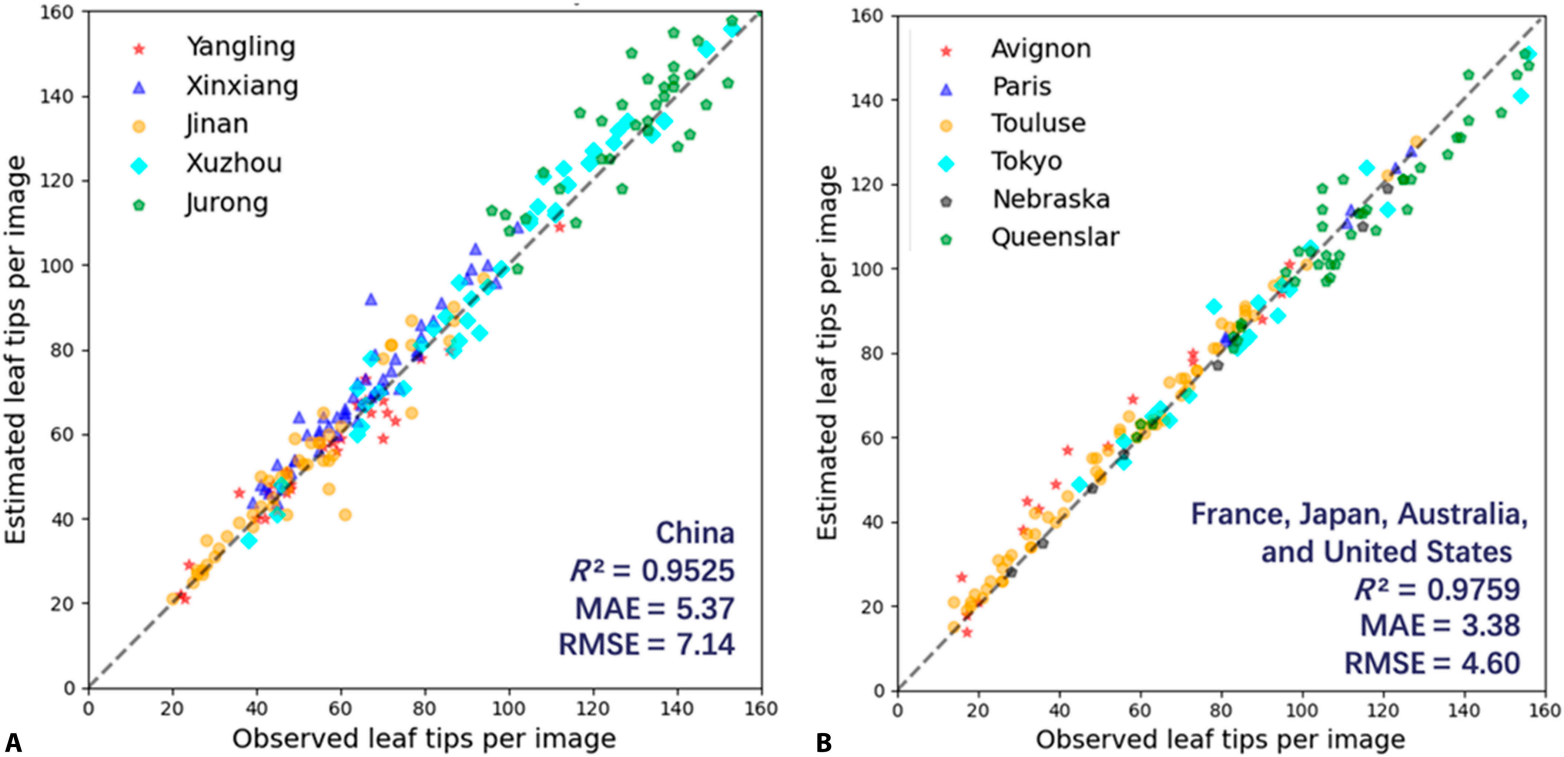
Prediction accuracy of the leaf counting model. (A) The distribution of all the sites where the Chinese dataset was collected. For each site, the results of leaf counting were visualized with red points on the leaf tip; (B) the prediction accuracy of the leaf counting model, CG-FRCNN, over the worldwide (France, Japan, Australia, and United States) dataset.

The robustness of the CG-FRCNN model was further evaluated using the 6 independent sites distributed across 4 countries (Table 1). Results show that the estimation of leaf tip counting was very accurate for all the sites and all the growth stages considered (Fig. 7B). Performances are very consistent with those reported for Chinese test dataset alone (Fig. 7A), with even slightly higher *R*^2^ and lower RMSE. The performance degrades marginally when the number of leaf tips is high, corresponding to around Haun stage 4. The CG-FRCNN model trained over the Sim2Real dataset therefore appears very robust across all the wide range of conditions represented by the 11 sites available. Note that the dataset used here is fully independent from the test dataset used to compare the model performance in Table 1. Moreover, the resolution of images used in this further evaluation is slightly higher than that in the preliminary test. This can explain the better counting accuracy (*R*^2^ = 0.97) than the preliminary test (*R*^2^ = 0.94).

## Discussion

### Novelty and efficiency of the proposed method

The capacity of characterizing crop traits under field conditions is highly desired by plant breeders who need to compare the performances of the genotypes under realistic environments [52,53]. We developed deep learning methods for detecting leaf tips of wheat from RGB images taken under field conditions. In a related study, Vishal et al. [23] used YOLOv3 model to detect the leaf tips of rice under greenhouse conditions. Field conditions are generally more difficult as compared to greenhouse-controlled conditions since the illumination conditions and the background may be very variable. However, our method proved to be efficient under a large range of field conditions.

While deep learning methods are considered as a breakthrough for the interpretation of high-resolution images, their success heavily depends on the size and representativeness of the training dataset. Supervised learning often assumes that both the training and test data come from the same distribution [42,54]. It requires a representative and accurate training dataset that often means a large quantity with a rich variation. However, this is not always achievable with dataset collected from the field and labeled manually. First, under field conditions, the variation of the canopy structure and the soil background (sometimes with weeds) aggregates the variation of image contents. This causes very large and diverse images required to be representative. Second, the labeling task especially for small objects is very time-demanding. Last, the manual labeling of images may be also prone to errors. Fortunately, some recent platforms such as D3P simulate very realistic RGB images while providing automatic labeling of the desired traits [44]. The resulting generation of simulated RGB images and the associated automatic leaf tip labels allowed building a much larger and more diverse training dataset. Unfortunately, the detection and counting performances were still very poor because of the significant domain shift that separates the simulated RGB images from the actual ones as we visualized by the t-SNE method.

Deep domain adaptation has emerged as a new learning paradigm to address the challenges above. We used simulated and real images to train domain adaption techniques. Among the several techniques, CycleGAN appears very efficient and allows us to get a much better match between the source (actual images) and the adapted simulated images (called Sim2Real here) as shown with the t-SNE method. Among the 6 deep learning models and adaptation techniques considered, the combination of CycleGAN and FRCNN provided the best performances for leaf tip detection and counting with *R*^2^ > 0.88 as observed across 4 Haun stages and a wide range of conditions.

The resulting proposed method appears very attractive since it eliminates the tedious, expensive, and sometimes inaccurate labeling task by simulating images for which the labels are automatically generated, while the realism of the images was improved using domain adaptation techniques. Such a combination of models to simulate images and domain adaptation techniques to improve their realism appears to be an innovative approach that has not yet been reported in the plant phenotyping community. It could be applied to a large number of other phenotyping problems.

### Limitations of the approach

The leaf counting is based on the area covered by each individual image. The proposed method should thus include the knowledge of the corresponding ground spatial resolution to get the leaf density, i.e., number of leaves per unit area. Although domain adaptation techniques allow improving the realism of the simulated images, it is necessary to provide images that are already sufficiently realistic enough as a basis. The ablation study that we conducted shows that the realism of the background, leaf texture, and illumination conditions impacts the performances of the detection model. Further, we demonstrated that the spatial resolution impacts the performance of the leaf tip detection, requiring the resolutions better than 0.6 mm per pixel.

The accuracy of the method tends to degrade as the Haun stage progresses. Even so, up to Haun stage 4 when tillering is just starting, the counting performances are still good. This is explained by the increased difficulty of finding tips in a more complex environment and by the fact that some tips may be masked by overlapping leaves. For later stages (after Haun stage 4) with a higher density of leaf tips, regression techniques may be more efficient than detection ones as demonstrated by Velumani [55] in the case of wheat head counting.

### Importance of the approach without human annotations

With the development of deep learning, great strides have been made in the accuracy of plant phenotype resolution. However, trained models often have poor generalization capabilities when used in completely novel scenarios. For this reason, large-scale commercial plant phenotyping is difficult. We found that the reason for this is the lack of large-scale training sets of real data with annotations. However, annotating large-scale real data is often time-consuming and laborious. Therefore, in recent years, the field of computer science has started to focus on training with large synthetic datasets to achieve generalizable model building [56].

We propose a more practical application of the plant phenotype resolution task, i.e., how to train models that generalize to unknown scenarios C using a large-scale labeled synthetic dataset A and an unlabeled real dataset B. This task no longer relies on manual annotation of real data and can therefore be extended to larger and more diverse real data, thus improving the generalization ability of the model. The method is claimed to be “self-supervised” since no manual labeling is necessary for the training process. It can learn well from labeled virtual data and unlabeled real data. Experiments show that this method, which does not require any manual annotation, is comparable to methods that require manual annotation in terms of generalization ability. It is a more promising and cost-effective solution among approaches to achieving plant phenotype resolution.

### Interests for physiological and genetic research

The growth potential of wheat seedlings, partly associated with the early vigor, reflects the gene–environment interactions [57,58]. The early vigor is characterized by a large leaf development at a given (early) stage. While the leaf area index may be accurately estimated using indirect methods [59], it can be decomposed into the number of leaves per unit area and the average leaf area. This decomposition may provide a better handle for the breeders to associate genes with these more detailed traits. Another trait that may be derived from the leaf tip counting is the phyllochron, defined as the time interval between the appearances of 2 consecutive leaves. The monitoring of the leaf tip number using fixed high-resolution RGB cameras or near-daily observations using any vehicle will allow estimating this trait and better understand its variation with the environment and the genome. This will be the objective of the next study.

## Data Availability

The data could be given upon reasonable request from the corresponding author.
